# A comprehensive assessment of lifecourse and mortality of Parkinson’s disease in the German National Cohort

**DOI:** 10.1038/s41531-026-01446-0

**Published:** 2026-06-26

**Authors:** Klaus Berger, André Karch, Christina M. Lill, Tom L. Berger, Ulrich Mueller, Michael Bartl, Brit Mollenhauer, Beate Fischer, Karin B. Michels, Philipp T. Meyer, Verena A. Katzke, Jara Sabin, Stefan N. Willich, Thomas Keil, Stefanie Castell, Anja M. Hauri, Matthias B. Schulze, Tamara Schikowski, Oliver Riedel, Tobias Pischon, Ilais Moreno Velasquez, Sebastian Heinzel, Hermann Brenner, Annette Peters, Rafael Mikolajczyk, Theresa Herrmann, Claudia Trenkwalder

**Affiliations:** 1 Institute of Epidemiology and Social Medicine, University of, Münster, Germany; 2 Ageing Epidemiology Research Unit (AGE), School of Public Health, Imperial College London, London, UK; 3 Institute for Health Services Research and Clinical Epidemiology, Medical School, Philipps-University, Marburg, Germany; 4 Department of Neurology, University Medical Center, Goettingen, Germany; 5 Paracelsus-Elena Klinik, Center for Parkinsonism and Movement Disorders, Kassel, Germany; 6 Institute for Epidemiology and Preventive Medicine, University of Regensburg, Bavaria, Germany; 7 Institute for Prevention and Cancer Epidemiology, Faculty of Medicine and Medical Center, University of Freiburg, Freiburg, Germany; 8 Department of Nuclear Medicine, Medical Center – University of Freiburg, Faculty of Medicine, University of Freiburg, Freiburg, Germany; 9 Division of Cancer Epidemiology C020, German Cancer Research Center (DKFZ), Heidelberg, Germany; 10 Institute of Social Medicine, Epidemiology and Health Economics, Charité-Universitätsmedizin Berlin, Berlin, Germany; 11 Institute of Clinical Epidemiology and Biometry, University of Wuerzburg, Wuerzburg, Germany; 12 State Institute of Health I, Bavarian Health and Food Safety Authority, Erlangen, Germany; 13 Department for Epidemiology, Helmholtz Centre for Infection Research, Brunswick, Germany; 14 Department of Molecular Epidemiology, German Institute of Human Nutrition Potsdam-Rehbruecke, Nuthetal, Germany; 15 Institute of Nutritional Science, University of Potsdam, Nuthetal, Germany; 16 IUF-Leibniz Institut für umweltmedizinische Forschung, Düsseldorf, Germany; 17 Leibniz Institute for Prevention Research and Epidemiology-BIPS, Bremen, Germany; 18 Molecular Epidemiology Research Group, Max-Delbrueck-Center for Molecular Medicine in the Helmholtz Association (MDC), Berlin, Germany; 19 Biobank Technology Platform, Max-Delbrueck-Center for Molecular Medicine in the Helmholtz Association (MDC), Berlin, Germany; 20 Charité - Universitätsmedizin Berlin, Corporate member of Freie Universität Berlin and Humboldt-Universität zu Berlin, Berlin, Germany; 21 Department of Neurology, Kiel University, University Medical Centre Schleswig-Holstein, Kiel, Germany; 22 Division of Clinical Epidemiology and Aging Research, German Cancer Research Center (DKFZ), Heidelberg, Germany; 23 Institute of Epidemiology, Helmholtz Zentrum München, German Research Center for Environmental Health, Neuherberg, Munich Germany; 24 Institute for Medical Information Processing, Biometry and Epidemiology (IBE), Faculty of Medicine, LMU Munich, Munich, Germany; 25 Institute for Medical Epidemiology, Biometrics, and Informatics, Interdisciplinary Center for Health Sciences, Medical Faculty of the Martin Luther University Halle-Wittenberg, Halle(Saale), Germany; 26 Dept. Neurosurgery, University Medical Center, Goettingen, Germany

**Keywords:** Diseases, Health care, Medical research, Neurology, Neuroscience, Risk factors

## Abstract

A strong increase in the prevalence and deaths of Parkinson’s disease (PD) has been reported over the last three decades. Ageing societies, genetic and environmental factors and access to health care contribute to these increases. Using data of the German National Cohort (NAKO) PD classifications based on self-report, claims data and a brief medication based algorithm to increase validity in the absence of neurologic examinations, were compared and the PD incidence, selected non-motor symptoms, functions and vital status were analysed. The algorithm based PD prevalence among 205.000 participants was 0.13% but it was 2.5-fold higher in claims data. Self-reported PD incidence was 0.13% over a median of 4.8 years follow-up, but cases had a 4-fold higher mortality, worse emotional, motoric and olfactory functions and higher prevalences of diabetes, obesity, depression and anxiety. The algorithm based PD prevalence and the mortality risk was similar to considerable older population studies from Europe.

## Introduction

The diagnosis of Parkinson’s disease (PD) is based on clinical symptoms since it was first described by James Parkinson^1^. While neuropathologic changes with Lewy Bodies in certain brain regions have later found to be a hallmark of the disease, the most frequently used UK Parkinson’s Disease Society Brain Bank Diagnostic Criteria and the MDS clinical definition criteria for PD^2^ include only clinical symptoms assessed in a neurologic examination. Multiple causes, including genetic and environmental factors, as well as toxicity and inflammation, can contribute to disease onset, but no consensus that reflects the complexity of the disease has been reached until now^3^.

Numerous publications in recent years have shown an increase in the absolute number of Parkinson patients due to demographic changes and also an increase in the number of PD patients who were affected by environmental risk factors, in particular toxins from agriculture, air pollution and specific fat-soluble toxins^4–13^. The ‘Global Burden of Disease’ project (GBD) found that PD was the neurological disorder with the strongest increase in prevalence, disability, and deaths, with a doubling of PD cases between 1990 and 2015 and an increase in mortality^14^. But, a recent review^15^ confirmed. differences in these numbers between countries, even within one region such as Europe, and concluded that regional variations in genetic plus environmental factors and differences in access to health care, regional life expectancy and study methods may contribute to the PD prevalence and mortality variations. Thus, there is a need for more detailed analyses of regional differences in health behaviors, environmental exposures and comorbidities accumulating over the life course plus variations in mortality.

While comparisons in disease frequencies across nations or regions can best be done by results of population based studies with a clear sampling frame, these data remain scarce. Thus, in large collaborative reviews such as the GBD Study, different data sources and results from various study designs are used, including claims data, death certificates, patient registers and cohort studies. While overlapping to a larger extent each data source has its methodological advantages and limitations and addresses PD frequencies and subsequent analyses from a slightly different viewpoint. In very large scale population-based cohort studies a classification of PD can usually not be based on a detailed neurologic examination and rather relies either on ICD codes (International Classification of Diseases), e.g., from claims data, or a (self-) reported physician diagnosis or a prescription of PD specific drugs or a combination of these factors.

The German National Cohort (NAKO) is a very large population-based study and provides a unique opportunity to examine prevalence, incidence, mortality as well as risk factors and comorbidity profiles for PD cases in a central European region. The aim of this analysis was to compare the PD frequency from different sources within the same population, to provide a brief algorithm to classify PD cases and to evaluate selected non-motor symptoms, comorbidities, functions and mortality related to PD status.

## Results

### PD classification

**Fig. 1:**
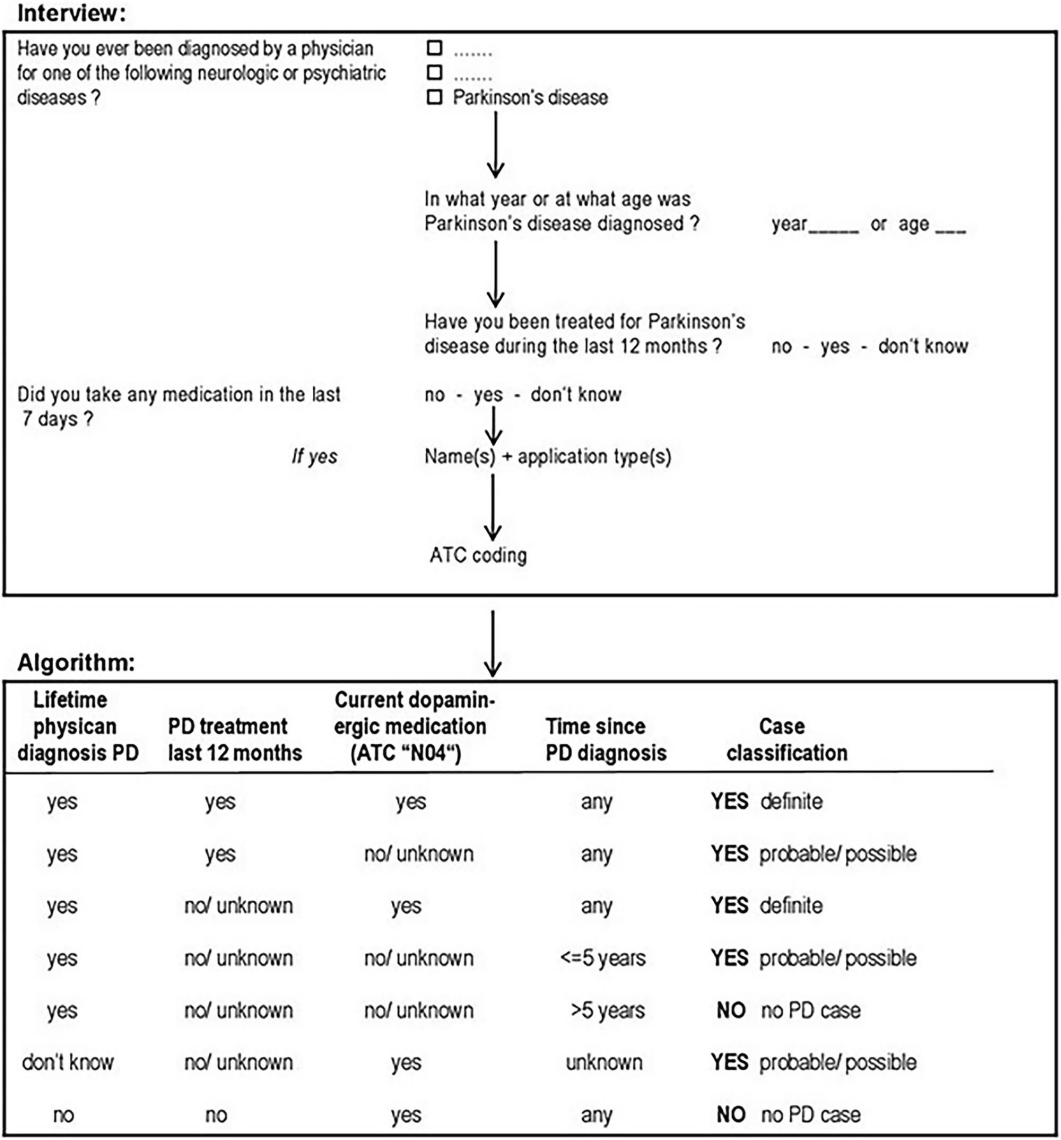
Parkinson’s disease classification algorithm at baseline in the German National Cohort, based on a self-reported diagnosis, time since diagnosis, current treatment status and medication in face-to-face interviews.

In Fig. 1 the PD classification algorithm is presented. Overall it resulted in 272 PD cases among the more than 204.000 NAKO participants, 228 classified as definite and 44 as probable/possible cases (Table 1). The PD diagnosis was made between three and six years prior to the baseline examination on average. 42 PD cases had died, on average (median) four years after their baseline examination, yielding an age and sex adjusted 4.2-fold higher risk (Odds ratio) of death, for all PD cases compared to non-cases. The time from PD diagnosis to death among deceased cases varied from a median of 16 years among those diagnosed before 50 years of age, to 14 years (diagnosed between 50 and 59 years) and 8 years among those receiving a physician-based PD diagnosis in age 60 years or higher. PD cases reported a higher proportion of a positive family history of PD, both paternal as well as maternal PD compared to non-cases, and, given their higher mean age compared to non-cases, a higher proportion of deceased parents.

**Table 1:** Frequency of algorithm based Parkinson’s disease classification, mortality and family history of Parkinson’s disease among NAKO participants

Total baseline participants, *N* (%)	204,273	(100.0)		
Classified as Parkinson’s disease, *N* (%)	272	(0.13)		
Definite case, *N* (%)	228	(0.11)		
Probable/possible case, *N* (%)	44	(0.02)		
Years since PD diagnosis				
Definite case, mean years	6.15			
Probable/possible case, mean years	3.72			
Mortality^a^				
*N* dead, % deceased and (age at death)				
Non-cases, *N*, *%*, (mean age)		3823	1*.87*	(64.97)
Definite PD, *N*, %, (mean age)		37	16.23	(71.95)
Probable/ possible PD, *N*, %, (mean age)		5	11.36	(67.60)
Risk of death, OR^b^ (95% CI)				
No PD	1.0	Ref.		
Definite PD	4.4	(3.0–6.3)		
Probable/possible PD	3.3	(1.3-8.6)		
Parental history of PD^c^				
History of PD for father				
No PD, %	2.6			
Definite PD, %	6.3			
Probable/possible PD, %	6.9			
History of PD for mother				
No PD, (%)	1.6			
Definite PD, (%)	5.4			
Probable/possible PD, (%)	3.7			
Father: % deceased and mean age at death (yrs)				
No PD, % dead (mean age)	50.5	(69.2)		
Definite PD, % dead (mean age)	81.1	(72.1)		
Probable/possible PD, % dead, (mean age)	80.7	(70.8)		
Mother: % deceased and mean age at death (yrs)				
No PD, % dead (mean age)	33.8	(72.8)		
Definite PD, % dead (mean age)	67.7	(77.4)		
Probable/possible PD, % dead (mean age)	70.0	(79.5)		
Incidence of self-reported PD diagnosis^d^, *N* (%)	196	(0.13)		
Among men, *N* (%)	121	(0.17)		
Among women, *N* (%)	75	(0.10)		

^a^Complete mortality follow-up until May 2022 plus deaths that became newly known until May 2024.

^b^Risk expressed as Odds Ratio plus 95% confidence interval of dying after the baseline examination until the end of 1st follow-up, adjusted for age in years and sex.

^c^Restricted to 177,980 participants with an available family history of disease (including 194 PD cases).

^d^Incidence among 149.473 participants (138,068 personal, 11,405 by written follow-up, 73,032 men and 76,441 women, follow-up response 73.2%) with non-missing answers to the medical interview questions and over a mean follow-up time of 4.8 years.

### PD prevalence

**Fig. 2:**
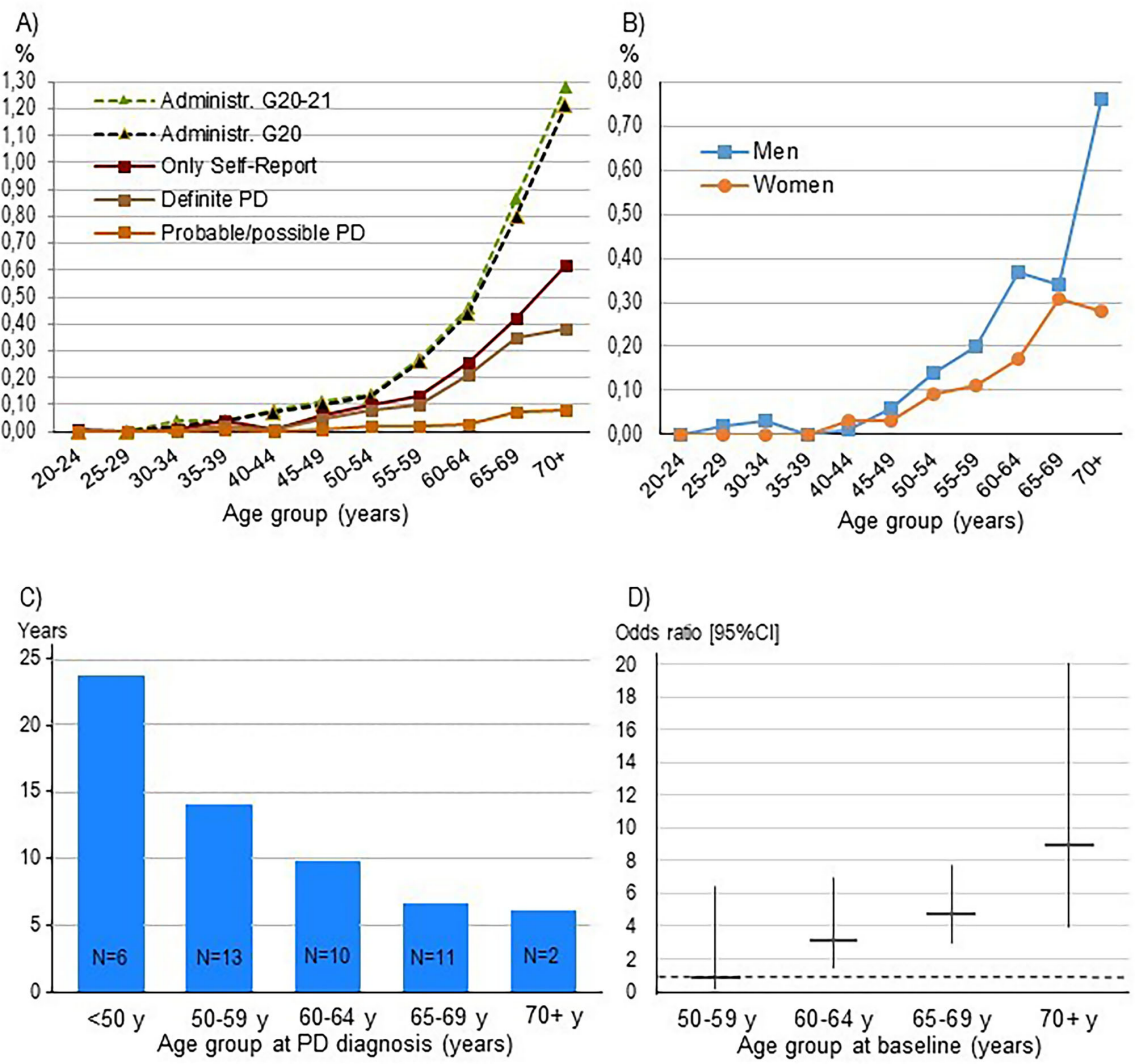
Frequencies of PD by different assessment methods and PD mortality risk. **A** Baseline prevalence (%) of a self-reported diagnosis of Parkinson’s Disease (dark red, *N* = 288), and of a definite (brown, *N* = 228) and probable/possible PD diagnosis (light brown, *N* = 44) according to the algorithm in Fig. 1 among all (*N* = 204,273) NAKO participants. In addition a G20/G21 (green dotted, *N* = 225) and a G20 only (black dotted, *N* = 208) ICD-10 Code in claims data of the subset with reimbursement data (*N* = 85,750) is shown. **B** Incidence (%) of a self-reported physician diagnosis of Parkinson’s Disease (*N* = 160) during an average 4.8 years follow-up (*N* = 138,063) according to sex and age group at baseline. **C** Mean time to death in years according to age at PD diagnosis. **D** Risk of death (expressed as odds ratio and 95% confidence interval) of PD cases according to age at baseline and adjusted for age (within age group) and sex, dotted line marks an OR of 1.0.

In Fig. 2a prevalences according to different methods of PD case classification and age groups are illustrated. The figure demonstrates that a claims data classification based on ICD-10 code ‘G20’ yields about two times higher prevalences than an exclusive self-report and about 2.5 times higher ones than the algorithm based classification into definite and probable/possible PD. Restricted to those with claims data the overall prevalence of an ICD-10 code “G20” was 0.24% and of a “G21” code 0.02%. The prevalence of a self-reported PD diagnosis 0.13% and the algorithm based prevalence 0.11%.

In Fig. 2b the incidence of a self reported PD diagnosis during an average of 4.8 years is plotted according to sex. 138.068 participants returned for the in-person first follow-up (Fup) examination and additional 11.405 filled in a Fup-questionnaire, yielding an overall response of 73.2%. The overall PD incidence based on self-reports was 0.13% over this follow-up period and differed significantly between men and women (men 0.17%, women 0.10%).

In Fig. 2c the mean number of years to death among deceased PD cases is visualized according to their age group at PD diagnosis. Finally, Fig. 2d shows the risk of death for PD cases within each age group compared to non-cases in the same group.

### PD case characteristics

Table 2 reports sociodemographic and clinical characteristics of the prevalent PD cases, classified by the algorithm, and non-cases at baseline, adjusted for age and sex. Cases were clearly older and more often male. While only a slight difference was observed in education, a large difference was found in the need of support in instrumental activities of daily living. While some of the differences in the comorbidity profiles were explained by age and/or sex, considerable differences with higher frequencies in the PD group were observed for obesity, diabetes, depression and anxiety. With regard to lifestyle factors, a lower proportion of PD cases consumed alcohol and were current smokers. 88% of the PD cases reported treatment with anti-parkinsonian drugs (ATC-code: N04) in the ‚last 7-days‘ medication assessment, with 42% of those treated being on a combination therapy with levo-dopa and a dopamine-agonist (ATC codes N04BA and N04BC).

**Table 2:** Sociodemographic characteristics and comorbidities of German National Cohort (NAKO) study participants at baseline, according to definite/probable PD status

				Unadjusted				Adjusted^a^ forage and sex	
				No PD			PD	No PD	PD
				Diagnosis			Diagnosis	Diagnosis	Diagnosis
				*N* = 204,001			*N* = 272	*N* = 204,001	*N* = 272
Age, median (years)				51.0			64.0	na	na
Age group 20–45 y, %				30.8			1.8	na	na
Age group 60–69 y, %				26.8			68.5	na	na
Women, %				50.5			39.0	na	na
Instrumental activities of daily									
living (max. 8 activities)^b^,% [95% CI]									
Needs in 1+ activities, %	42.3	[41.8–42.7]	73.7		[65.6–80.5]*	42.1	[41.6–42.6]	73.0	[64.7–80.0]*
Mean number of needs (max. 8)	2.3	[2.3–2.4]	3.8		[3.5–4.2]*	2.3	[2.3–2.4]	3.9	[3.6–4.2]*
among those with needs									
ISCED-97 level^c^ high, % [95% CI]	55.1	[54.9–55.3]	58.5		[52.2–64.4]	55.2	[54.9–55.4]	59.1	[52.9–65.1]
Comorbidities^d^, % [95% CI]									
Myocardial infarction	1.7	[1.6–1.7]	3.7		[2.0–6.7]	0.7	[0.6–0.7]	0.6	[0.3–1.1]
Cardiac dysrhythmia	9.1	[9.0–9.3]	17.7		[13.6–22.6]	8.1	[8.0–8.2]	10.1	[8.0–14.1]
Peripheral artery disease	1.8	[1.8–1.9]	8.6		[5.8–12.6]*	1.2	[1.1–1.2]	2.8	[1.8–4.3]*
Hypertension	27.6	[27.3–27.7]	47.5		[41.5–53.4]*	23.3	[23.0–23.4]	24.5	[20.1–29.3]*
Obesity (BMI ≥ 30 kg/m^2^)	21.0	[21.3–21.7]	36.6		[30.8–42.6]*	20.8	[20.6–21.0]	28.0	[23.2–33.5]*
Diabetes	6.0	[5.9–6.1]	15.4		[11.6–20.2]*	4.5	[4.4–4.6]	6.6	[4.8–9.0]**
Epilepsy	0.7	[0.7–0.7]	3.3		[1.7–6.2]*	0.8	[0.8–0.9]	3.1	[1.6–5.9]*
Depression	14.6	[14.5-14.8]	30.8		[26.0–37.0]*	14.0	[13.8–14.2]	28.8	[23.7–24.4]*
Anxiety or panic disorder	7.9	[7.8–8.0]	14.3		[10.7–19.0]*	7.5	[7.4–7.6]	13.5	[10.0–18.0]*
Lifestyle factors, %/ m [95% CI]									
Current alcohol consumption, %	91.4	[91.3–91.6]	79.6		[73.9-84.3]*	91.5	[91.4-91.6]	79.8	[74.2–84.5]*
Mean gram alcohol/day drinkers	11.6	[11.5–11.7]	13.9		[11.4-16.4]	11.6	[11.5–11.7]	11.3	[8.9–13.8]
Smoking status, % [95% CI]									
Never	46.2	[46.0–46.4]	49.4		[43.1–55.7]	46.1	[45.9–46.4]	54.5	[48.2–60.8]**
Current	20.5	[20.3–20.7]	12.1		[8.5–16.9]*	20.3	[20.1–20.4]	13.9	[9.9–19.3]**

^a^Adjusted for age (in years) and sex (men, women).

^b^Restricted to age group 60–69 years only (*N* = 43,901 with 133 PD cases).

^c^ISCED = International Standard Classification of Education 1997.

^d^Self-reported lifetime diagnosis by a physician.

*p ≤ 0.002, ** *p* ≤ 0.02.

^na^Not calculated.

In Table 3 different non-motor and motor functions are summarized, after adjustment for age, sex and education. PD cases reported higher means or proportions in all scales, especially in the domains of current depressive symptoms and isolation. Considerable differences were measured in motoric and olfactory functions. Hand grip strength differed by about 4 kg after adjustments, while the olfactory screening test revealed that only 43% of the PD cases where normosmic, compared to 85% among non-cases. Subjective memory complaints were clearly higher in the PD group, especially among individuals that perceived memory changes with sorrow. In contrast, only small differences were measured in the different cognitive function tests, most pronounced in the Stroop test, a test of executive function, with slightly longer times measured in the PD group.

**Table 3:** Motor and non-motor functions in NAKO participants at baseline, according to PD status and adjusted for age, sex and education

	No PD		PD	
	Diagnosis		Diagnosis	
	*N* = 204,001		*N* = 272	
Emotional functions^a^				
PHQ-9 summary score, mean	3.9	[3.9–4.0]	6.9	[6.4–7.4]*
M.I.N.I. Screen-positive, %	27.9	[27.7–28.1]	32.1	[26.8–37.9]
GAD-7 summary score, mean	3.2	[3.2–3.2]	4.5	[4.1–5.0]*
PHQ-Stress summary score, mean	3.5	[3.5–3.6]	4.8	[4.4–5.3]*
Childhood-Trauma summary score, mean	7.3	[7.2–7.3]	8.0	[7.6–8.4]*
Social-Network-Index: Isolated, %*	13.4	[13.3–13.6]	16.1	[11.1–22.8]
Motor functions^a^				
Hand grip strength kg, mean	39.2	[39.1–39.2]	35.9	[35.0–36.8]*
L2 Purdue Peg Board^c^, mean	9.9	[9.9–9.9]	7.8	[7.2–8.3]*
L2 Olfactory function *^c^ (Sniffin-Sticks-12)				
Normosmia, %	85.0	[84.6–85.3]	43.4	[29.4–58.5]*
Correct^a^ of smells (max.12), mean	10.3	[10.3–10.3]	7.2	[6.6–7.7]*
Subjective memory complaints^a^^,b^				
Subjective memory complaints, %	23.9	[23.6–24.1]	37.8	[32.1–43.7]*
Subj. memory complaints with sorrow, %	13.9	[13.7–14.0]	27.3	[22.2–33.1]*
Cognitive function tests^b^				
Animal names (60 s), mean	26.1	[26.1–26.1]	24.7	[23.9–25.6]*
Word list 1st recall (max. 12), mean	6.9	[6.9–6.9]	6.4	[6.2–6.6]*
Word list 2nd recall (max. 12), mean	9.6	[9.6–9.7]	9.0	[8.8–9.2]*
Word list 3rd recall (max. 12), mean	8.4	[8.4–8.4]	7.7	[7.4–7.9]*
Stroop board 2, seconds, mean	21.0	[21.0–21.0]	22.8	[22.3–23.3]*
Stroop board 3, seconds, mean	40.9	[40.8–40.9]	48.0	[46.0–49.1]*
Numbers series backward, mean	4.9	[4.9–4.9]	4.8	[4.6–5.0]

^**a**^Adjusted for age (in years), sex (men, women) and education (education years according to ISCED-97 classification). Instrument/scale acronyms see method section.

^b^Each test is adjusted for age (in years), sex (men, women) and education (education years according to ISCED-97 classification). Participants with moderate to poor knowledge of the German language and/or with potential problems in hearing, vision and/or external test disturbances were excluded.

^c^Restricted to L2 participants with the indepth examination program (max. *N* = 60,332 participants, including *N* = 61 PD cases).

^d^Restricted to age groups 45+ years (*N* = 140,071 participants including 263 PD cases).

*p ≤ 0.002, ** *p* ≤ 0.02.

## Discussion

Using data from a large population-based study in Germany we compared PD prevalences at baseline based on different classifications, self-reported diagnosis, diagnosis from claims data and a classification algorithm that takes self-reports, medication and duration into account. We additionally report PD incidences based on self-reports and compared mortality and non-motor and motor functions of PD the algorithm classified cases with non-cases. The algorithm-based prevalence was clearly lower than the claims data classification but was comparable to previous, considerably earlier conducted, population based studies^16^ in Europe. For the interpretation of the different PD classifications the underlying research questions must be considered. The claims data based prevalence primarily answers questions about healthcare utilization and related costs to a society that are summarized under a PD code. The ICD-Code G20 might therefore include multiple system atrophy type Parkinson or Lewy body dementia, progressive supranuclear palsy or even more rare syndromes at the time of diagnosis, or in the early years. But, the frequency of these differential diagnoses is very low. A merely self-reported diagnosis of PD reflects the diagnosis perception and/or knowledge of an individual. Here lack of knowledge or a misunderstanding of the diagnosis or even an indignation to share the information may occur and explain a gap between numbers. The algorithm-based classification combines more objective criteria, such as medication and treatment status, with the individual report, thereby increasing the validity of a PD status. While the personal examination by a movement disorder specialist with assessment of the diagnostic criteria of PD is the gold standard of case classification and, thus the determination of a diagnosis, this is often impossible to do in studies with a large number of participants due to limitation of resources, especially in so-called mega-cohorts such as NAKO or the UK Biobank Study. In this situation a classification that combines several criteria that can be easily assessed, improves the case classification compared to self-reports alone.

Using a completely different design a recent study from the Danish Parkinson registry^17^ observed similar prevalences as the NAKO algorithm based one. In that study PD cases in routinely collected health registry data of adults were identified either from the Danish National Patient (DNP) registry or the Danish Prescription Medicines (DPM) registry. Those identified were asked to confirm their PD diagnosis using a national self-report survey. This study also combined self-reported diagnosis and (prescriptions of) medication, however, subsequently compared them with national health registers not readily available in Germany.

The only other population based study^18^ in Germany, conducted almost 30 years ago among 982 community dwelling elderly (65+ years) in southern Germany using a door-to-door examination design, reported a PD prevalence of 0.7% and of 1% for parkinsonism of other cause. In NAKO with its more than 200,000 participants across the country a detailed neurologic examination was obviously not possible. Thus, the development of a diagnostic algorithm to classify PD cases is a feasible alternative but might over- or underestimate the population PD frequency. Overestimation of the PD prevalence might occur by inclusion of other non-PD parkinsonian syndromes. Underestimation could be based on the cohort design, which requires patients to visit the study center for an extensive and exhausting examination, which might be difficult for disabled PD patients. Definite as well as probable/possible cases in this setting followed a clear age pattern with a steep increase after the age of 60 years until the limited upper age range in the study. This pattern is in line with results from the GBD Study^14,19^ and its details for European countries. But the latter study also reported a considerable increase in PD over 30 years, when comparing 1991 to 2021^19^.

The GBD Study described an increase in overall PD mortality between 1990 and 2016 of 9% in Western Europe and of 14.5% for Germany. Compared to non-cases we observed considerably higher mortality risk among cases in NAKO, even higher than reported in prior population based studies from Europe^20^. This might be due to higher risks of PD complications among those affected in young age. This message may change future clinical prognosis communications with patients and families, especially when the diagnosis is uncertain in prodromal stages or even non-clinical stages^21^.

Many prior reports have confirmed higher scores in current depressive and anxiety symptoms including lifetime histories of depression and anxiety disorder among PD cases. But the higher proportions of diabetes and obesity observed in NAKO PD cases, support the need for more detailed analyses of risk factors in the development of PD. Prior studies showed that elevated blood pressure and other cardiovascular and metabolic risk factors increase the severity of PD^22^, as does a specific cardiovascular risk profile detected with plasma biomarkers^23^; while the use of antihypertensive drugs, when adjusted for “arterial hypertension” and “renal disease”, appears to reduce the PD incidence^24^. The differences in olfactory and motor functions were expected and were previously confirmed. The considerable difference in subjective memory complaints is noteworthy, given the only slight differences between cases and non-cases observed in the scores of the neuropsychological test battery.

This study has several strengths and limitations. Among the strengths are the large sample size, its sample frame in the general population due to the random participant selection from local community registries and the highly standardized assessment in all study centers based on standard operating procedures (SOPs) and trained and certified study nurses. The availability of claims data at baseline and follow-up data for the same individuals are further strengths. Among the limitations is the fact that detailed neurologic examinations or specific PD scales for a case classification could not be performed, given the sample size. In the NAKO setting of a population-based study with a very large number of participants the purpose of our analysis was rather to apply a method that increases the validity of a self-reported PD diagnosis, in the absence of the infrastructure and resources needed to apply the gold standard of a clinical examination. Thus, we aimed at an improvement of the validity of self-reports for PD, knowing that a certain proportion of misclassification, e.g., individuals who don’t (yet) know about a PD diagnosis or very early cases who are not treated and differential diagnoses such as Parkinson syndromes due to vascular lesions or atypical PD, cannot be excluded. Future improvements of the algorithm might include more detailed medication information on treatment combinations as well as an integration of blood-based biomarkers. The age range of the participants invited for the baseline examination (20–69 years) excluded the ‚high-risk by age‘ groups since PD prevalence increases strongly with age. But because follow-up examinations are ongoing future analyses will be able to use the 5- and 10-year follow-up incidences of PD. In addition, the relative low overall response in NAKO of about 17%^25,26^ in combination with a certain healthy participant bias that is common in all population-based studies, might lead to selection and an underestimation of PD frequencies, since more severe and disabled PD cases, especially in case of cognitive impairments, are less likely to attend the (baseline) examination.

In summary, we provide a comprehensive description of frequencies, functional status and mortality of PD in a large population based study, comparing different classifications of disease and resulting prevalences. We provide a short PD classification algorithm, that is easy to apply in large cohort studies. The observed considerably increased risk of mortality among cases in all age groups has a potential to influence counseling of affected families in the future.

## Methods

The German National Cohort (NAKO) is one of the worldwide few so-called mega-cohorts^27^ and recruited more than 205,000 individuals in 18 study centers across Germany between 2014 and 2019^25^. Participants, aged 20 to 69 years, were randomly selected within 5-year age and sex strata in the local community registries (age groups 20 to 39 were slightly underweighted) and invited to participate. In most study centers the random sample that was subsequently invited to the study, was only drawn once at the local city registries. Therefore a small proportion (*N* = 3.948, 1.9%) of the participants was 70 years and older (max.74 y) at the time of first examination. The overall response was 17 percent for the baseline survey^25^. The standard examination program (level 1, L1) included a computer assisted face-to-face medical interview, several clinical-diagnostic examinations, measurements and tests, a touchscreen based self-report part and a comprehensive biomaterial collection. A random 25% sample of the participants in all centers was assigned to an intensified program (level 2, L2) that included additional in-person examinations and tests. The standard program L1 lasted on average four hours, the intensified L2 program 5.5 h. The German National Cohort is conducted in accordance with the Declaration of Helsinki and each participant had signed the informed consent. The ethics committee of the Bavarian Chamber of Physicians, as the lead ethic committee, had approved the study (Nr. 13023) and this vote was adopted by the committees of each local study center.

### Socioeconomic status and lifestyle factors

Details of education, partnership, occupational and economic situation were asked in the face-to-face interview. Education was subsequently classified according to the International Standard Classification of Education 97 (ISCED-97)^28^ into education years. Different lifestyle factors were included in the touchscreen based self-reports, among them a detailed assessment of the smoking history and current smoking status. Consumption of alcohol was based on type of drink, amount and frequency and subsequently calculated as gram alcohol per day. Instrumental activities of daily living (IADL) assessment was done with the 8-item IADL part of the Lawton and Brody Scale^29^, also as part of the self-report questions, but restricted to age-groups 60+ years. The proportion of elder participants with needs in one or more of the 8 activities and the mean number of needs among those with needs were subsequently calculated.

### Diseases, medication and mortality

In the interview participants were asked for lifetime diagnoses by a physician for a large number of diseases, grouped into six disease groups, including PD. In case of any diagnosis the time point (age or year) was assessed and if this condition was treated within the last 12 months. Current medications, taken within the last seven days, were collected and directly coded according to the anatomical-therapeutical-chemical code (ATC). For the family history a list of 10 diseases, including PD, was documented in the touchscreen self-report, separately for mother and father as well as their current vital status and age at death if deceased. Restless legs syndrome (RLS) cases were classified according to the so-called minimal criteria of the International Restless Legs Study Group IRLSSG^30^, among participants receiving the L2-examination program only. NAKO assesses the vital status of the study participants on a regular basis in a dedicated mortality-follow-up unit located at the Federal Institute for Population Research (BiB). The unit asks for the vital status of those 15.000 participants with no contact for at least 2 years in a rolling way twice per year. For the present analysis the baseline data from 2014 to 2019, the new incidence of a physician-based PD diagnosis from the first follow-up (2019 to 2024) and the complete vital status of the entire cohort until May 2022 plus all newly known deaths as of May 29th 2024 (end of 1st follow-up) was used. For a subset of 85.750 participants health insurance (claims) data were additionally available. Health insurance is mandatory in Germany. ICD-10 codes G20 (Parkinson Disease) and G21 (Secondary parkinsonism), documented twice within 12 months (M2Q criteria) plus a ‘confirmed’ coding in diagnostic certainty, were used in an alternative way to classify cases.

### PD algorithm

The classification algorithm aimed to achieve a PD case group with higher diagnostic accuracy compared to self-report alone, given the setting of a megacohort. A ‘definite’ classification by the algorithm (Fig.1) refers to the situation that a participant is positive for a physician-based PD diagnosis, treatment for PD during the last 12 months, a current dopaminergic therapy based on ATC codes and a plausible disease duration. Accordingly probable/possible cases have missing information to a varying degree for one or more of these four factors resulting in more uncertainty of a PD classification. The few individuals who gave a ‘don’t know’ answer to the PD diagnosis question, but took a documented dopaminergic therapy were included, unless they also reported a Restless Legs Syndrome (RLS).

### Emotional functions

A lifetime history of depression, anxiety and panic disorder was assessed in the medical interview as listed above. In addition, the module on major depressive disorder (MDD) of the structured Mini International Neuropsychiatric Interview German version 5.0.0 (MINI)^31^ was used in the interview. L1 participants were asked the two screening questions of the MDD module, while only L2 participants who screened positive, received the additional MDD module questions that allow a MINI based depression diagnosis^32^. Touchscreen self-reports included current depressive symptoms during the last two weeks, based on the Patient-Health-Questionnaire depression scale (PHQ-9)^33^ and anxiety symptoms during the last 4 weeks using the Generalized-Anxiety-Disorder-Screener (GAD-7)^34^ and for current stress symptoms the psychosocial stressor module of the Patient-Health-Questionnaire (PHQ-10)^35^. A brief screening version of the Childhood Trauma Questionnaire, the 5-item CT-S^36^, was applied to assess trauma in childhood and youth. The Social Network Index (SNI)^37^ was employed for participants aged 45+ years to assess different categories of relationships, including partnership, friends and family, memberships in other formal or informal groups. The SNI provides four categories of increasing social connections with the lowest category indicating ‚isolation‘. For the PHQ-9, GAD-7, PHQ-10 and CT-S scales the respective summary scores were calculated.

### Olfactory function

The assessment of olfactory function was restricted to L2 participants and used the Sniffin-Sticks-12 (Burghart Messtechnik GmbH, Holm, Germany) forced choice odor identification test. It allows a categorization of participants into anosmic, hyposmic, and normosmic functions, based on the identification of 12 different odors. Normosmia was defined as 10 to 12 correctly recognized sticks, hyposmia as 7–9 and anosmia as less than 7 correct sticks, according to the manual. An adaptation was made for stick 6, for which prior analysis^38^ has shown that one of the three distractors had the same recognition frequency as the correct smell and therefore two correct smells were accepted.

### Motoric functions

Motoric function was examined by two tests. Handgrip strength was measured three times for each hand using the Jamar+ device (Sammons Preston, Rolyon, Bolingbrook, IL, USA) and the maximum grip strength in kilogram was used in the analyses. Fine motor movements, only examined in L2 participants, were measured with the Purdue Peg Board^39^.

### Cognitive functions

The NAKO baseline examination included a neuropsychologic test battery^40^ applied by trained and certified study assistants. The battery addressed different cognitive domains and was based on six tasks from four established tests, including semantic fluency (animal names), verbal episodic memory (10 nouns from the CERAD wordlist^41^ plus 2 nouns with same word frequency in German), processing speed and executive control (Stroop Colored Word Test^42^) and verbal working memory (digit span backwards). Subjective memory complaints were assessed by five questions as part of the medical interview. They addressed the subjective perception of potential memory problems and if such problems were self-perceived as worrisome (sorrow) and if a physician contact had already taken place because of this concern.

### Statistics

The interpretation of results from large cohort studies such as NAKO has to take the very high sample size into account. Given the more than 204,000 NAKO baseline participants any small difference between two groups has a high likelihood to be statistically significant, irrespective of clinical or functional relevance. Therefore, results in Table 1 were restricted to the description of means, medians and proportions. For the calculation of the odds ratio of mortality a logistic regression model was applied with death as the dependent and age, sex and algorithm-based PD status as the independent variables. For the analysis of sociodemographic and clinical factors in Table 2 unadjusted means and proportions, including their 95% confidence intervals, and in addition age and sex adjusted values, are presented, since both factors are related to PD status. For the functional results shown in Table 3 only age (in years), sex (men, women) and education (ISCED-97 education years) adjusted means and proportions are presented, since all these functions are, to a varying degree, influenced by these factors. For these calculations the adjusted means (based on linear regression) and adjusted proportion (based on logistic regression) procedures from the STATA 17 statistical package were used.

## Data Availability

Access to and use of NAKO data and biosamples can be obtained via an electronic application portal (https://transfer.nako.de) that provides details about the application process.
